# Comment on “Association of Ticagrelor Metabolic SNPs With Adverse Drug Reactions in Patients With Acute Coronary Syndrome”

**DOI:** 10.1002/clc.70428

**Published:** 2026-07-29

**Authors:** Yanyan Bai, Cheng Liu, Liuliu Feng

**Affiliations:** ^1^ Department of Cardiology Shidong Hospital Shanghai China

Dear Editor,

We read with great interest the recent study by Zhang et al. on the association between CYP3A4/5 genetic polymorphisms and adverse drug reactions to ticagrelor [1]. This prospective study established a strong link between the CYP3A5 rs776746 CC genotype and ticagrelor‐related dyspnea, offering a potential genetic tool for precision medicine. However, we believe several aspects warrant further discussion.

First, the statistical analysis in this study did not correct for multiple hypothesis testing. The simultaneous examination of associations between two single‐nucleotide polymorphisms (rs776746, rs2242480) and multiple clinical endpoints (dyspnea, bleeding, revascularization, mortality) significantly increases the risk of obtaining false‐positive results (Type I error) by chance. In genetic association studies, correction for multiple testing is a standard procedure to ensure the robustness of conclusions [2]. Some of the reported *p* values in the article are borderline significant and may lose statistical significance if relatively conservative methods such as Bonferroni correction or false discovery rate (FDR) control are applied. Therefore, in the absence of validation in an independent cohort, interpretation of the current findings should be approached with caution.

Second, the definition of the primary endpoint, dyspnea, in the study is overly broad, potentially masking important clinical heterogeneity. The study defined an endpoint event as Common Terminology Criteria for Adverse Events (CTCAE) grade ≥ 1, employing a binary classification that fails to distinguish events of differing clinical significance. Evidence from large‐scale clinical trials indicates that ticagrelor‐associated dyspnea is typically mild, transient, and in most cases does not affect long‐term prognosis or treatment adherence [3]. The events of critical clinical importance are moderate‐to‐severe dyspnea (grade 2 and above) that lead to reduced patient adherence or unplanned treatment discontinuation. Aggregating all grades of dyspnea for statistical analysis may dilute the predictive specificity of this genotype for the severe adverse reactions that are of genuine clinical concern. Future research should employ more granular endpoint stratification and integrate patient‐reported outcomes such as the proportion of dyspnea leading to treatment discontinuation and symptom duration, to enhance the clinical applicability of predictive models.

Finally, the study failed to evaluate the impact of genotypes on net clinical benefit, which may result in an imbalanced risk assessment. The core endpoints of the study focused solely on adverse drug reactions (dyspnea and bleeding), overlooking the influence of genotypes on drug efficacy—specifically, the prevention of ischemic events such as stent thrombosis or recurrent myocardial infarction. Clinical decision‐making is essentially a dynamic balance between ischemic and side‐effect risks [4]. If the CYP3A4 rs2242480 T allele reduces bleeding risk by lowering the exposure level of the active metabolite, a direct consequence may be a weakened antiplatelet effect. In the absence of simultaneous assessment of ischemic endpoints such as myocardial infarction or stent thrombosis, genotyping based solely on adverse reaction risks may lead to one‐sided treatment decisions and potentially compromise the overall prognosis of patients.

In summary, although the study by Zhang et al. provides new insights into predicting ticagrelor‐related adverse events, future research must be grounded in more rigorous statistical standards, more refined clinical phenotyping, and comprehensive evaluation of net clinical benefit.

## Author Contributions

**Yanyan Bai:** conceptualization, writing – original draft. **Cheng Liu:** conceptualization, writing – original draft. **Liuliu Feng:** writing – review and editing, supervision, validation.

## Ethics Statement

The authors have nothing to report.

## Consent

The authors have nothing to report.

## Conflicts of Interest

The authors declare no conflicts of interest.

## Data Availability

Data sharing is not applicable to this article as no data sets were generated or analyzed during the current study.
